# Body composition phenotypes and phase angle as predictors of mortality in subjects with right ventricular dysfunction: a prospective cohort study

**DOI:** 10.3389/fnut.2026.1952746

**Published:** 2026-09-22

**Authors:** Dulce González-Islas, Arturo Orea-Tejeda, Ana Gabriela Meléndez-Galeana, Andrea Bárcenas-Montiel, Danna Paola Jiménez-Cruz, Juan David De la Borda-Paredes, Estrella Vargas-Corona, Sergio Martin Alday-Ramirez, José Carlos Ruan-Díaz, Benigno Valderrábano-Salas, Laura Flores-Cisneros

**Affiliations:** 1 Servicio de Cardiología, Instituto Nacional de Enfermedades Respiratorias Ismael Cosío Villegas, Ciudad de México, Mexico; 2 Dirección General de Epidemiología, Secretaría de Salud, Ciudad de México, Mexico

**Keywords:** cachexia, dynapenia, exercise tolerance, mortality, phase angle, right ventricular dysfunction, sarcopenia

## Abstract

**Background:**

Right ventricular dysfunction (RVD) is characterized by abnormal RV structure or function and is associated with worse outcomes. In subjects with RVD, cardiac cachexia and sarcopenia were associated with intestinal congestion. However, the prognostic impact and prevalence of body composition phenotypes and phase angle (PhA) in RVD have been insufficiently studied.

**Objective:**

To assess the prognostic impact and prevalence of body composition phenotypes and PhA in RVD,

**Methods:**

A prospective cohort study was conducted. Outpatient subjects over 18 years old with a confirmed diagnosis of RVD were included. Dynapenia, muscle wasting, and sarcopenia, according to the European Working Group on Sarcopenia in Older People 2 (EWGSOP2), while cachexia was defined according to Evans criteria.

**Results:**

A total of 212 subjects with RVD were included. The mean age of the subjects was 64.47 ± 13.99 years, and 58.96% were men. The prevalence of dynapenia was 28.30%, muscle wasting was 10.38, sarcopenia was 21.70%, and cachexia was 4.25%. The independent risk factors for mortality were PhA < 5° (HR: 2.29, 95% CI: 1.27 to 4.15, *p* = 0.006), low performance (HR:2.69, 95% CI: 1.25 to 5.81, *p* = 0.011), sarcopenia (HR: 2.58, 95% CI 1.05 to 6.34; *p* = 0.038), and cachexia (HR: 4.29, 95% CI 1.29 to 14.24; *p* = 0.017).

**Conclusion:**

In subjects with RVD, there is a higher prevalence of muscle disorders such as dynapenia, sarcopenia, and cachexia. Besides PhA, sarcopenia and cachexia were associated with a worse prognosis.

## Background

RVD is defined as abnormal RV structure or function and is associated with worse outcomes, independent of the underlying disease mechanisms (1). Generally, heart failure (HF) and impairments in skeletal muscle mass and function share common pathophysiological mechanisms, including chronic inflammation, hormonal changes, endothelial dysfunction, insulin resistance, low muscle blood flow, impaired muscular oxygen uptake, mitochondrial dysfunction, and oxidative stress (2, 3).

In RVD, increased right atrial pressure and decreased stroke volume activate the renin-angiotensin-aldosterone system and cause systemic venous congestion. This congestion causes intestinal hypoperfusion, promoting increased translocation of bacterial lipopolysaccharide and production of pro-inflammatory cytokines, as well as anorexia and malabsorption. These factors trigger activation of catabolic pathways and inhibition of anabolic muscle pathways, worsening mass and muscle strength (4, 5).

In subjects with RVD, Valentova et al. (5) demonstrated that cardiac cachexia and sarcopenia were associated with intestinal congestion, independent of HF stage and cardiac function. They found that these patients experienced a higher prevalence of post-prandial fullness, appetite loss, and abdominal discomfort, as well as increased bowel thickness in the entire colon and terminal ileum.

In HF subjects, the predominant body composition alterations include fluid status and low phase angle (PhA), besides body composition phenotypes such as muscle wasting, low handgrip strength, also known as dynapenia, sarcopenia, obesity, and cachexia, all of which have been linked to worse clinical outcomes (5–8).

The PhA parameter derived from bioelectrical impedance analysis (BIA) is a useful indicator of membrane integrity and cell health. A low PhA suggests lower cellularity, membrane integrity, cellular function, and malnutrition status. In addition, PhA is associated with a worse prognosis across diverse populations (9, 10).

On the other hand, sarcopenia is defined as a progressive, generalized loss of skeletal muscle mass, strength, and function. It is associated with an increased likelihood of adverse outcomes in diverse populations, including falls, fractures, disability, loss of independence in daily living activities (IADLs), hospitalization, and mortality (7, 9, 10). In subjects with concomitant left-sided HF and sarcopenia, more pronounced impairments in mobility, muscle strength, endurance, and IADLs have been observed (10).

In left-sided HF, the prevalence of sarcopenia was found to be 31%. In individuals younger than 65 years, it was 25%, increasing to 31% in those older than 65 years. Regarding HF type, sarcopenia was found in 18% of heart failure preserved ejection fraction (HFpEF) cases and 28% of heart failure reduced ejection fraction (HFrEF) cases (7). In terms of prognosis, left-sided HF is associated with a risk of poor outcomes (HR: 1.64, 95% CI: 1.20 to 5.25). However, when analyzed by type of HF, only subjects with HFrEF showed a significant association with worse prognosis (HR: 2.77, 95% CI: 1.29 to 5.95), while this was not the case for HFpEF (HR: 1.61, 95%CI: 0.82 to 3.16) (7). Respect to cachexia is a complex catabolic syndrome characterized by unintentional edema-free weight loss. Cachexia results from complex mechanisms, such as neurohormonal dysregulation, inflammation, and gastrointestinal dysfunction, among others (11). Cachexia is an independent predictor for increased all-cause mortality (8). Despite this, the prevalence and prognostic impact of musculoskeletal disorders in RVD remain insufficiently studied. Our primary objective was to assess the prognostic impact of body composition phenotypes and PhA; our secondary objective was to determine the prevalence of these conditions in subjects with RVD.

## Methods

A prospective cohort study was conducted at the Instituto Nacional de Enfermedades Respiratorias Ismael Cosío Villegas in Mexico City, Mexico, from December 1, 2019, to May 31, 2025.

Outpatient subjects over 18 years old with a confirmed diagnosis of RVD, according to the European Society of Cardiology (12, 13), were included. Diagnosis was based on tricuspid annular plane systolic excursion < 17 mm, fractional area change < 35%, and free lateral wall S′ < 9.5 cm/s at tissue Doppler imaging. Patients with cancer, human immunodeficiency virus, neurological disease, or a history of hospitalization within the previous 3 months were excluded.

The study was conducted in accordance with the Declaration of Helsinki and approved by the Institutional Ethics Committee of the Instituto Nacional de Enfermedades Respiratorias “Ismael Cosío Villegas” (Project Approval Number E02-18)

### Data collection

Demographic, clinical, anthropometric, body composition, exercise tolerance, and echocardiographic variables were assessed at the beginning of the study cohort as part of the clinical management of outpatient patients at our institute, as previously described in Ibarra-Fernández et al. (14).

#### Anthropometry

Weight and height were measured according to the manual reference for anthropometric standardization (15). All subjects wore light clothing and were barefoot.

#### Handgrip strength

Handgrip strength was measured using a mechanical Smedley Hand Dynamometer (Stoelting, Wood Dale, UK) according to the technique described by Rodríguez-García et al. (16) Subjects stood with their arms parallel to the trunk, picked up the dynamometer, and applied maximum force with the dominant hand. To avoid fatigue, the measurement was repeated three times, with 1 min between each repetition. The maximum value was recorded in kilograms.

#### Bioelectrical impedance analysis (BIA)

Total body composition was measured using whole-body BIA with four-pole multifrequency equipment (BodyStat QuadScan 4,000, BodyStat, Isle of Man, UK) by standard technique (17). The BIA method injects an alternating electric current of minimal intensity below the sensing thresholds. The impedance (Z) represents the opposition that biological materials show to the passage of an alternating electric current. The electrical impedance Z comprises resistance (R) and reactance (Xc). The current passage determines R through the intracellular and extracellular electrolyte solutions, while Xc represents the delay in current flow, measured as a phase shift, reflecting the dielectric properties of cell mass and integrity of the cell membranes (18). The same operator conducted the measurements in the morning in a comfortable area free of drafts, with portable electric heaters. The area was cleaned before the study. Subjects were fasting and had not exercised for 8 h or consumed alcohol for 12 h prior to the study. During the study, subjects were positioned supine, with arms separated from the trunk by about 30° and legs by about 45°. Electrodes were placed on the hand and the ipsilateral foot.

PhA was calculated using the following equation: PhA (degrees) = arctan (Xc/R)·(180/π). In our population, the PhA at the 50th percentile was 5°.

The impedance ratio was calculated as the ratio of the high (200 kHz) to the low frequency (5 kHz) of multifrequency BIA (19). In our population, the impedance ratio at the 50th percentile was 0.84.

#### Appendicular skeletal muscle mass index (ASMMI)

ASMMI was assessed using Sergi’s equation (20): ASMMI (kg/m^2^) = [−3.964 + (0.227 * height^2^ (cm)/R) + (0.095 * weight) + (1.384 * sex) + (0.064 * Xc)/height (m^2^)].

#### Obesity

Obesity was defined as excess adiposity (fat mass ≥ 30% for men and ≥ 40% for women) (21).

#### Exercise tolerance

Exercise tolerance was assessed by 6MWD, performed according to American Thoracic Society standards (22).

#### Low performance

Low performance was defined as non-completion of the 6MWD test or a walk distance < 350 meters.

### Body composition phenotypes

Body composition phenotype was categorized as preserved strength and muscle mass, dynapenia, muscle wasting, sarcopenia, or cachexia. These categories were mutually exclusive, with each participant classified into only one phenotype category.

#### Preserved strength and muscle mass

Preserved strength and muscle mass was defined as normal handgrip strength (≥ 27 kg for men and ≥ 16 kg for women), normal ASMMI (≥ 7.0 kg/m^2^ for men and ≥ 6.0 kg/m^2^ for women) (9).

#### Dynapenia

Dynapenia was defined as low muscle strength according to EWGSOP2 as handgrip strength < 27 kg for men and < 16 kg for women (9).

#### Muscle wasting

Muscle wasting was defined as low muscle mass according to the European Working Group on Sarcopenia in Older People 2 (EWGSOP2) (9) as ASMMI < 7.0 kg/m^2^ for men and < 6.0 kg/m^2^ for women.

#### Sarcopenia

Sarcopenia was defined according to EWGSOP2 (9) as the presence of low muscle strength (< 27 kg for men and < 16 kg for women) and low muscle mass (ASMMI < 7.0 kg/m^2^ for men, and < 6.0 kg/m^2^ for women).

#### Cachexia

Cachexia was defined as body mass index (BMI) < 20 kg/m^2^ plus three or more of the following five features: decreased muscle strength, fatigue, anorexia, low fat-free mass index, and abnormal biochemistry (elevated inflammatory markers, anemia, or low albumin) (11).

### Endpoint

The endpoint was defined as all-cause mortality. Death records were obtained through referrals from relatives or registration in the hospital medical records.

### Statistical analysis

Analyses were conducted using STATA version 14 (Stata Corp., College Station, TX, USA). Categorical variables were expressed as frequencies and percentages. The Shapiro–Wilk test was used to assess the normality of continuous variables. Normal variables were expressed as mean and standard deviation, while non-normal variables were reported as median and percentiles 25–75. Comparisons between study groups (survival vs. non-survival) were analyzed using the chi-square test for categorical variables and the t-student test or Mann–Whitney U test for continuous variables.

Cox proportional-hazards regression models were used to assess the association between body composition and mortality. First, univariate Cox models were constructed to estimate the association between demographic, clinical, and body composition variables and mortality.

Subsequently, only body composition parameters with *p*-values <0.20 in the univariate analyses were included in the multivariable Cox regression analyses. To minimize potential multicollinearity among body composition measures, each selected parameter was evaluated in a separate multivariable Cox proportional-hazards model. Each model was adjusted for demographic and clinical variables with *p*-values <0.20 in the univariate analyses, including sex, age, obesity, and HF. Potential interactions and multicollinearity were systematically evaluated. As a sensitivity analysis, interactions between RVD etiology (left-sided HF or COPD) and body composition parameters were formally tested using a joint Wald test. No significant interaction was identified between body composition phenotype and obesity. A complete-case analysis was performed for each model, excluding subjects with missing data on the covariates included in that specific model. Results are reported as hazard ratios (HR) with 95% confidence intervals (CI). Statistical significance was defined as a two-sided *p*-value <0.05.

## Results

Two hundred and twelve subjects with RVD were included. The median follow-up was 1,042 days (range 417.5–1872), and 27% of subjects died during the follow-up. The mean age of the subjects was 64.47 ± 13.99 years, and 58.96% were men. Non-surviving subjects were older and had lower prevalences of diabetes and obesity than surviving subjects (Table 1).

**Table 1:** Clinical characteristics of right ventricular dysfunction subjects in surviving and non-surviving subjects.

Clinical characteristics	All *n* = 212	Non survival *n* = 58	Survival *n* = 154	*p*-value
Men, *n* (%)	125 (58.96)	35 (60.34)	90 (58.44)	0.802
Age, years	64.47 ± 13.99	71.39 ± 11.16	61.86 ± 14.08	<0.001
Comorbidities				
Hypertension, *n* (%)	109 (51.42)	77 (50.00)	32 (55.17)	0.502
COPD, *n* (%)	78 (36.79)	25 (43.10)	53 (34.42)	0.242
CKD, *n* (%)	30 (14.15)	7 (12.07)	23 (14.94)	0.594
Diabetes, *n* (%)	84 (39.62)	16 (27.59)	68 (44.16)	0.028
Asthma, *n* (%)	9 (4.25)	3 (5.17)	6 (3.90)	0.708
Heart failure, *n* (%)	144 (67.92)	41 (70.69)	103 (66.88)	0.597
Heart failure type, *n* (%)				
HFrEF	44 (20.75)	10 (17.24)	34 (22.08)	0.533
HFmrEF	15 (7.08)	3 (5.17)	12 (7.79)	
HFpEF	85 (40.09)	28 (48.28)	57 (37.01)	
Echocardiographic parameters				
LVEF, %	54.10 ± 15.53	56.00 ± 14.83	53.42 ± 15.77	0.300
TAPSE, mm	17 [15–19.15]	17 [15–18]	17 [15.5–20]	0.177
Systolic S velocity of tricuspid annulus, cm/seg	9.5 [8.8–10.4]	9.5 [9–10]	9.5 [8.7–10.7]	0.795
Fractional area change, %	37.10 ± 9.79	36.11 ± 10.49	37.46 ± 9.53	0.396
Body composition parameters				
Weight, kg	74.15 ± 20.69	66.98 ± 24.10	76.85 ± 18.64	0.006
Height, cm	159.63 ± 10.07	158.57 ± 10.31	160.02 ± 9.98	0.352
BMI, kg/m^2^	29.17 ± 8.31	26.46 ± 8.16	30.20 ± 8.16	0.003
Obesity, *n* (%)	117 (55.19)	23 (39.66)	94 (61.04)	0.005
Handgrip strength, kg	22.86 ± 9.24	19.72 ± 8.76	24.04 ± 9.16	0.002
Phase angle, °	5.19 ± 1.28	4.64 ± 1.10	5.39 ± 1.29	<0.001
TBW, %	51.99 ± 8.56	54.15 ± 8.48	51.12 ± 8.48	0.047
ECW, %	23.1 [21.6–25.6]	24 [22.2–27]	22.7 [21.3–25.2]	0.037
ICW, %	29.4 [25.2–31.6]	29.4 [24.9–31.40]	29.4 [25.2–31.6]	0.502
Impedance index, 200/5 kHz	0.83 [0.80–0.86]	0.84 [0.81–0.87]	0.83 [0.80–0.85]	0.074
Fat mass, %	34.88 ± 10.17	30.88 ± 10.14	35.23 ± 9.96	0.005
ASMMI, kg/m^2^	7.13 ± 1.25	6.73 ± 1.42	7.28 ± 1.15	0.004
6MWD, meters	325.46 ± 144.36	257.05 ± 150.66	350.34 ± 134.33	<0.001
Low performance, *n* (%)	87 (64.44)	29 (80.56)	58 (58.59)	0.018
Body composition phenotype				
Preserved strength and muscle mass, *n* (%)	75 (35.38)	9 (15.52)	66 (42.86)	<0.001
Dynapenia, *n* (%)	60 (28.30)	18 (31.03)	42 (27.27)	
Muscle wasting, *n* (%)	22 (10.38)	4 (6.90)	18 (11.69)	
Sarcopenia, *n* (%)	46 (21.70)	21 (36.21)	25 (16.23)	
Cachexia, *n* (%)	9 (4.25)	6 (10.34)	3 (1.95)	

COPD, chronic obstructive pulmonary disease; CKD, chronic kidney disease; HFrEF, heart failure with reduced ejection fraction; HFmrEF, heart failure with mildly reduced ejection fraction; HFpEF, heart failure with preserved ejection fraction; LVEF, left ventricular ejection fraction; TAPSE, tricuspid annular plane systolic excursion; BMI, body mass index; TBW, total body water; ECW, extracellular water; ICW, intracellular water; ASMMI, appendicular skeletal muscle mass index; 6MWD, 6-min walk distance.

Regarding body composition parameters, non-surviving subjects had lower weight, BMI, handgrip strength, PhA, fat mass, ASMMI, and 6MWD, as well as higher total body water and extracellular water, and a higher prevalence of low performance.

With respect to body composition phenotypes in RVD subjects, non-survival subjects had a higher prevalence of dynapenia (31.03% vs. 27.27%), sarcopenia (36.21% vs. 16.23%), and cachexia (10.34% vs. 1.95%) than survival subjects (Table 1).

Figure 1 illustrates the distribution of body composition phenotypes with and without obesity. Among the subjects, 35.38% had preserved strength and muscle mass, of whom 9.91% were not obese and 25.47% were obese; 28.30% had dynapenia, of whom 8.96% were not obese and 19.34% had dynapenic obesity; 10.38% had muscle wasting, of whom 7.08% were not obese and 3.77% were obese; 21.70% had sarcopenia, of whom 14.64% were not obese and 6.60% had sarcopenic obesity; and 4.25% had cachexia.

**Figure 1:**
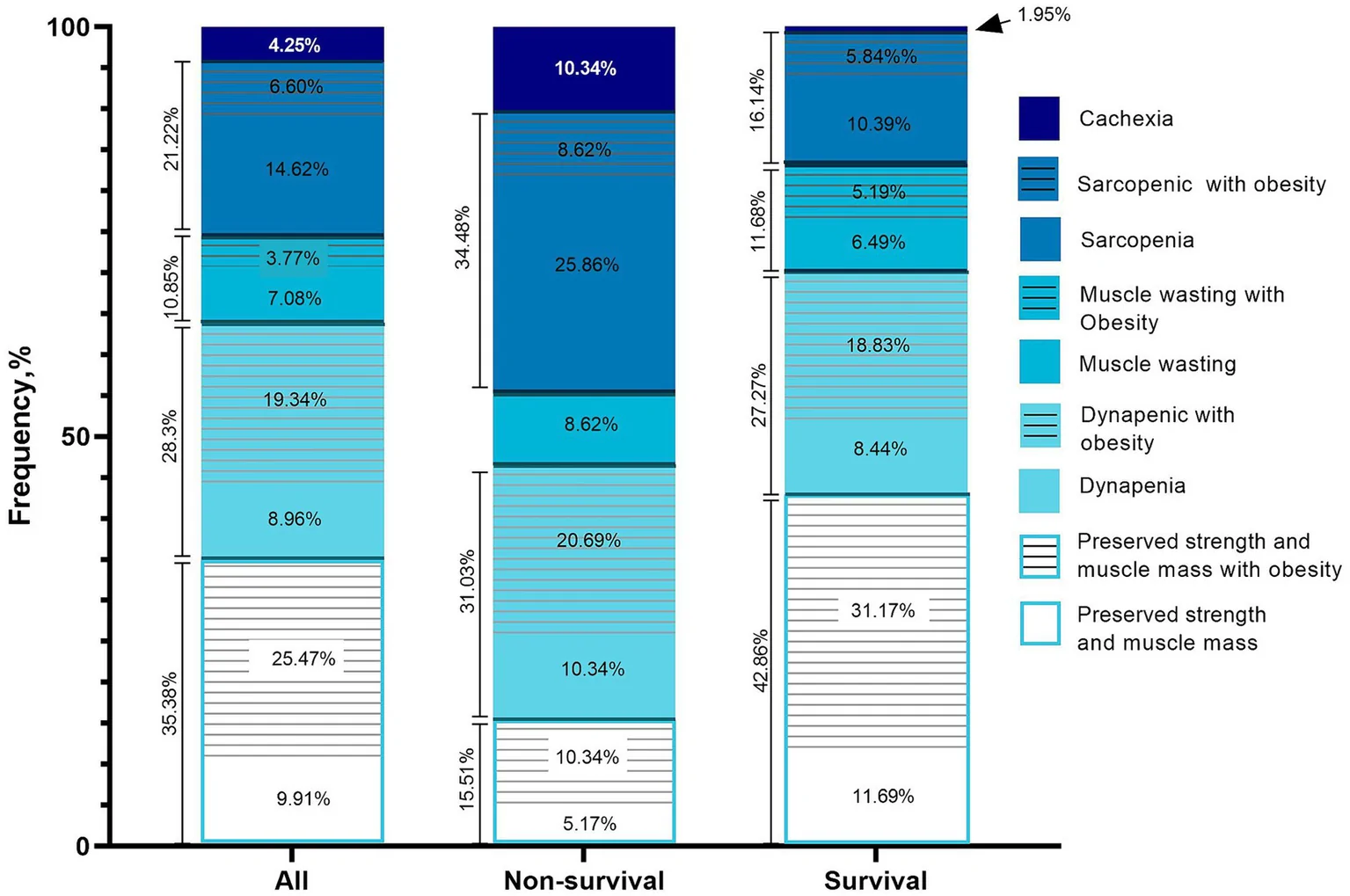
Body composition phenotypes with and without obesity.

In the univariate model, age, HFpEF, PhA < 5°, impedance ratio > 0.84, and low performance were associated with a higher risk of mortality, while obesity, weight, BMI, handgrip strength, fat mass, ASMMI, and 6MWD were associated with a lower risk of mortality in subjects with RVD. With respect to body composition phenotypes, dynapenia, sarcopenia, and cachexia were associated with a higher risk of mortality (Table 2).

**Table 2:** Univariate analysis of body composition predictors of mortality in RVD subjects.

Predictors of mortality	HR	CI 95%	*p* value
Men	1.40	0.82 to 2.39	0.207
Age, years	1.04	1.02 to 1.07	<0.001
Comorbidities			
Hypertension	1.21	0.72 to 2.03	0.463
COPD	0.87	0.51 to 1.49	0.627
CKD	1.14	0.51 to 2.54	0.743
Diabetes	0.68	0.38 to 1.22	0.203
Asthma	1.37	0.43 to 4.41	0.588
Heart failure	1.73	0.97 to 3.08	0.062
Heart failure type			
HFrEF	1.65	0.74 to 3.66	0.216
HFmrEF	0.91	0.26 to 3.12	0.887
HFpEF	1.97	1.06 to 3.65	0.030
Body composition parameters			
Weight, kg	0.98	0.96 to 0.99	0.004
Height, cm	1.00	0.97 to 1.02	0.798
BMI	0.93	0.90 to 0.97	0.001
Obesity	0.43	0.25 to 0.73	0.002
Handgrip strength, kg	0.96	0.93 to 0.99	0.015
Phase angle < 5 °	2.85	1.63 to 4.97	<0.001
TBW, %	1.03	0.99 to 1.06	0.064
ECW, %	1.01	0.97 to 1.05	0.502
ICW, %	1.04	0.96 to 1.12	0.283
Impedance index >0.84	1.86	1.00 to 3.48	0.050
Fat mass, %	0.96	0.93 to 0.98	0.002
ASMMI	0.73	0.58 to 0.91	0.006
6MWD, meters	0.99	0.99 to 0.99	<0.001
Low performance	2.95	1.44 to 6.00	0.003
Body composition phenotypes			
Preserved strength and muscle mass, *n* (%)	1	Reference	
Dynapenia, *n* (%)	2.41	1.08 to 5.37	0.031
Muscle wasting, *n* (%)	1.73	0.53 to 5.64	0.359
Sarcopenia, *n* (%)	4.66	2.13 to 10.19	<0.001
Cachexia, *n* (%)	6.09	2.16 to 17.15	0.001

COPD, chronic obstructive pulmonary disease; CKD, chronic kidney disease; HFrEF, heart failure with reduced ejection fraction; HFmrEF, heart failure with mildly reduced ejection fraction; HFpEF, heart failure with preserved ejection fraction; BMI, body mass index; TBW, total body water; ECW, extracellular water; ICW, intracellular water; ASMMI, appendicular skeletal muscle mass index; 6MWD, 6-min walk distance.

Multivariate analysis showed that PhA < 5° (HR: 2.29, 95% CI: 1.27 to 4.15, *p* = 0.006), low performance (HR: 2.69, 95% CI: 1.25 to 5.81, *p* = 0.011), sarcopenia (HR: 2.58, 95% CI 1.05 to 6.34; *p* = 0.038), and cachexia (HR: 4.29, 95% CI 1.29 to 14.24; *p* = 0.017) were associated with a higher risk of mortality after adjusting for confounding variables (Table 3).

**Table 3:** Multivariate analysis of Body composition predictors of mortality in RVD subjects.

Body composition parameters	HR	CI 95%	*p* value
Weight, kg	0.99	0.97 to 1.01	0.524
BMI, kg/m^2^	0.96	0.89 to 1.03	0.279
Obesity	0.78	0.41 to 1.47	0.453
Handgrip strength, kg	0.94	0.90 to 0.98	0.009
Phase angle < 5	2.29	1.27 to 4.15	0.006
TBW, %	1.01	0.94 to 1.10	0.626
Impedance index >0.84	1.74	0.89 to 3.42	0.105
ASMMI, kg/m^2^	0.82	0.62 to 1.10	0.195
6MWD, meters	0.99	0.99 to 0.99	0.001
Low performance	2.69	1.25 to 5.81	0.011
Body composition phenotypes			
Preserved strength and muscle mass, *n* (%)	1	Reference	
Dynapenia, *n* (%)	1.86	0.81 to 4.28	0.139
Muscle wasting, *n* (%)	2.11	0.61 to 7.20	0.233
Sarcopenia, *n* (%)	2.58	1.05 to 6.34	0.038
Cachexia, *n* (%)	4.29	1.29 to 14.24	0.017

TBW, Total body water; ASMMI, Appendicular skeletal muscle mass index; 6MWD, 6-min walk distance.

## Discussion

The primary finding of our study highlights the impact of PhA, sarcopenia, and cachexia on prognosis in subjects with RVD. It also shows the high prevalence of body composition alterations in RVD.

In this study, 28.30% of subjects exhibited dynapenia. Muscle strength was assessed using handgrip strength, which correlates with overall muscle strength (23). Muscle strength is recognized as a robust biomarker of overall health, functional capacity, ability to perform activities of daily living, and mortality risk in various populations (24–26). It is also associated with all-cause mortality in left-sided HF (27). However, most research on muscle strength in HF has focused on left-sided HF, while RVD remains understudied. In this study, dynapenia was associated with mortality in the univariate model but not in the multivariate model. This discrepancy may be attributable to sample size or limited follow-up duration.

Muscle strength is a better predictor of adverse outcomes and prognosis than muscle mass (23). One reason muscle strength may be a better predictor than muscle mass is that strength is lost before muscle mass. In older adults, the loss of muscular strength has been shown to be 3–4% per year, while the loss of lean mass is about 1% per year (28). Furthermore, an increase in adipose tissue, particularly intermuscular fat, has been observed independent of changes in body weight (29). This infiltration of fat into skeletal muscle, termed myosteatosis, induces inflammation, mitochondrial dysfunction, oxidative stress, and promotes insulin resistance, leading to a vicious cycle of muscle mass loss and increased myosteatosis (30). Moreover, muscle strength serves as an indicator of functional and quality muscle (31). It is more sensitive to the structural and metabolic changes that occur in skeletal muscle, such as myosteatosis (31).

Regarding low muscle mass, our study found a prevalence of muscle wasting of 10.38%, but no association with mortality in RVD. In left-sided HF, von Haehling et al. (32) observed a prevalence of 17% in left-sided HF subjects; 21.5% in HFrEF and 9.2% in HFpEF. A recent meta-analysis in left-sided HF subjects indicated that low muscle mass was associated with increased mortality (33).

As previously mentioned, there are interconnected mechanisms between HF and impairments in skeletal muscle mass and function. The reduction in tissue perfusion occurs in both the myocardium and peripheral tissues, triggering hypoxia, pro-inflammatory cytokines, and oxidative stress, which also promote skeletal muscle apoptosis. Additionally, the loss of muscle mass activates signaling pathways that mediate muscle inflammation, perpetuating a vicious cycle. Angiotensin II has an inhibitory effect on insulin-like growth factor-1, inducing mitochondrial dysfunction and fiber-type transition from type I to type II, contributing to muscle atrophy (34).

A meta-analysis by Chen et al. (7) reported a prevalence of sarcopenia in left-sided HF of 31%, with rates of 28% in HFrEF and 18% in HFpEF. However, the prevalence of sarcopenia in RVD has not been previously evaluated. In the present study, the prevalence of sarcopenia was 21.70% and was higher among non-surviving subjects. Subjects with sarcopenia demonstrated a 2.44-fold increased risk of mortality (HR: 2.58, 95% CI 1.05 to 6.34) after adjustment for confounding variables. Sarcopenia is characterized by decreased muscle strength and low muscle mass. In left-sided HF, sarcopenia is an independent predictor of hospitalization, adverse cardiovascular events, and mortality (7). Sarcopenia and HF share interconnected pathophysiological pathways, including a pro-inflammatory state, nutritional deficiencies, neurohormonal activation, hypercatabolism, reduced perfusion, and impaired oxygen delivery (35, 36).

Cachexia is a multifactorial syndrome characterized by severe body weight, fat, and muscle loss, along with increased protein catabolism due to underlying disease (37). Since muscle mass loss occurs before fat tissue loss, sarcopenia precedes cachexia (37). Cachexia is considered an advanced stage of myopathies, characterized by a more hypermetabolic state and inflammation than sarcopenia (37, 38). In HFrEF and HFpEF, the prevalence of cachexia was found to be 18.8% (39). In our study, the prevalence was 4.25%, and it was associated with a greater risk of mortality (HR: 4.29, 95% CI 1.29 to 14.24). Previous studies have proposed in subjects with an ejection fraction > 50%, Melenovsky et al. (40) observed that those with RVD experienced greater anorexia, edema, cachexia, pulmonary artery pressure, C-reactive protein, and B-type natriuretic peptide, along with lower fat mass compared to subjects without RVD. Additionally, RVD was associated with a greater risk of cachexia and adverse outcomes (40). This may be explained by the fact that RVD subjects experience venous congestion, which promotes intestinal congestion, leading to anorexia, malabsorption, and gut bacteria translocation that activates the catabolic and inhibit anabolic muscle pathways (4, 5).

In addition to this, subjects with HF are characterized by fatigue and exercise intolerance, which are associated with worse quality of life and an increased risk of hospitalization and mortality. In our study, 66.44% have low performance, and was associated with worse prognosis. Traditionally, exercise intolerance is attributed to cardiac dysfunction; however, sarcopenia and cachexia plays a significant role. In patients with HF, there is metabolic inefficiency, myocyte apoptosis, reduced muscle mass, and an increase in type 1 muscle fibers. (for aerobic metabolism) that are fatigue-resistant, a lower capillary-to-fiber ratio, and an increased proportion of IIx fibers have all been observed. These changes have been associated with lower exercise tolerance, as evaluated by VO2 peak (41, 42).

On the other hand, PhA is a raw BIA variable that reflects cellular health, cell membrane integrity, hydric status, and nutritional status. Higher PhA values indicate better cellular health, higher muscle mass and quality, better body water distribution, and a better prognosis (43, 44). In compensated and acute decompensated left-sided HF, a cut-off value of <4.68 and <4.8, respectively, of PhA was independently associated with increased mortality (45, 46). In our study, PhA < 5° was an independent predictor of mortality (HR: 2.29, 95% CI: 1.27 to 4.15, *p* = 0.006).

PhA values vary by age, sex, and population. However, in our population, no reference values are available. In our study, a PhA cutoff of < 5° was defined as the 50th percentile of our cohort. A study of patients with HF found that a PhA cutoff of 5° (sensitivity 0.84, specificity 0.80) was optimal for detecting congestion. In our study, we selected the median as our primary approach, given the relatively modest sample size of our cohort, which limited the statistical power to derive a stable ROC.

As the evidence shows, alterations in body composition play an important role in exercise tolerance, IADLs, quality of life, and prognosis in subjects with RVD. This highlights the importance of interventions targeting increased muscle quality, such as combined nutritional interventions and resistance-based exercise. By detecting these changes early, we can help prevent the onset of the disease.

### Strengths and limitations of the study

Among the limitations of this study are a single-center study and the moderate sample size. Another limitation of our study was that we were unable to determine the specific cause of death, particularly whether it was cardiovascular or respiratory. Cause-specific mortality was not systematically assessed in our cohort because death confirmation relied on family reports and hospital medical records rather than formal verification of the cause of death. Given that RVD can have both cardiac and pulmonary origins, cause-specific mortality could provide additional insight into the mechanisms underlying the prognostic value of body composition parameters. A complete-case analysis was used for each multivariable model, which assumes that data are missing completely at random. No missing data were present for the primary exposures of interest (phase angle and body composition phenotype). For secondary parameters, 6MWD had a higher proportion of missing data; missingness was not significantly associated with the prognostic outcome, providing some reassurance against outcome-dependent bias. However, we cannot fully rule out that missingness was related to unmeasured patient characteristics (missing at random or missing not at random), which could reduce statistical power or introduce bias in these exploratory models. Future studies employing multiple imputation methods may help further address this limitation. However, the strength of our study lies in its prospective cohort design, which allowed us to assess causality. We also used multivariate models, which enabled us to adjust for potential confounding variables. Additionally, this study explores the impact of body composition phenotypes on prognosis in subjects with RVD, which is crucial given that existing publications primarily focus on left-sided HF, but in RVD is limited.

## Conclusion

In subjects with RVD, there is a higher prevalence of muscle disorders such as dynapenia, sarcopenia, and cachexia. Besides PhA, sarcopenia and cachexia were associated with a worse prognosis.

## Data Availability

The raw data supporting the conclusions of this article will be made available by the authors, without undue reservation.
